# Automated sepsis detection with vancomycin- and allantoin-polydopamine magnetic nanoparticles

**DOI:** 10.1038/s41598-024-54236-0

**Published:** 2024-02-14

**Authors:** Abdurhaman Teyib Abafogi, Jinyeop Lee, Joochan Kim, Sei Won Lee, Seongsoo Jang, Sungsu Park

**Affiliations:** 1 School of Mechanical Engineering, Sungkyunkwan University, Suwon, 16419 Korea; 2 KingoBio Inc., Seoul, 08390 Korea; 3 Department of Pulmonology and Critical Care Medicine, Asan Medical Center, University of Ulsan College of Medicine, Seoul, 05505 Korea; 4 Department of Laboratory Medicine, Asan Medical Center, University of Ulsan College of Medicine, Seoul, 05505 Korea

**Keywords:** Sepsis, Molecular diagnostics, IMS, Polydopamine, Vancomycin, Allantoin, Automation, Analytical biochemistry, High-throughput screening, Lab-on-a-chip, Nanobiotechnology

## Abstract

Rapid and accurate identification of the bacteria responsible for sepsis is paramount for effective patient care. Molecular diagnostic methods, such as polymerase chain reaction (PCR), encounter challenges in sepsis due to inhibitory compounds in the blood, necessitating their removal for precise analysis. In this study we present an innovative approach that utilizes vancomycin (Van) and allantoin (Al)-conjugated polydopamine (PDA)-coated magnetic nanoparticles (MNPs) for the rapid and automated enrichment of bacteria and their DNA extraction from blood without inducing clumping and aggregation of blood. Al/Van-PDA-MNPs, facilitated by IMS, eliminate the need for preliminary sample treatments, providing a swift and efficient method for bacterial concentration and DNA extraction within an hour. Employing Al/Van-PDA-MNPs within an automated framework has markedly improved our ability to pre-concentrate various Gram-negative and Gram-positive bacteria directly from blood samples. This advancement has effectively reduced the detection threshold to 10^2^ colony-forming unit/mL by both PCR and quantitative PCR. The method's expedited processing time, combined with its precision, positions it as a feasible diagnostic tool for diverse healthcare settings, ranging from small clinics to large hospitals. Furthermore, the innovative application of nanoparticles for DNA extraction holds promising potential for advancing sepsis diagnostics, enabling earlier interventions and improving patient outcomes.

## Introduction

Sepsis, a critical medical condition resulting from bacterial infections in the bloodstream^1–4^, poses a significant threat as it can progress to organ failure and potentially lead to fatal outcome^4–8^. The underlying cause of sepsis lies in an uncontrolled immune response triggered by the presence of bacteria in the bloodstream, accompanied by the release of toxins from rapidly multiplying bacteria^1–3,5^. The urgency of timely diagnosis cannot be overstated, as the lack of appropriate intervention results in a 10% increase in mortality every hour^4,9,10^. Current diagnostic methods for sepsis, primarily reliant on microbiological blood examinations, are time-intensive, requiring anywhere from 24 h to several days depending on bacterial growth rates^11–13^. While gene amplification-based molecular diagnostics have emerged as a more rapid alternative^9,14^, their accuracy is frequently compromised by inhibitory compounds in blood^15,16^. Notably, elements such as heme, leukocyte DNA, and anticoagulants, including EDTA and heparin, diminish the effectiveness of molecular diagnostics ^15–17^. To elevate the sensitivity and precision of molecular diagnostic methods, it is crucial to eliminate these inhibitory compounds ^17^. This challenge underscores the pressing need for techniques that can effectively remove these compounds from blood samples, thereby increasing the reliability and sensitivity of molecular diagnostics for sepsis. Addressing these obstacles is imperative to establish a dependable and timely procedure for sepsis diagnostics.

In efforts to improve the sensitivity of molecular diagnostics, several commercialized kits and automated nucleic acid extraction systems have been developed ^18,19^. Extraction of nucleic acids from samples has been shown to enhance the sensitivity of PCR systems. However, commercially available extraction kits have limitations in terms of sensitivity improvement. These limitations are attributed to the small sample volume (200 µL) utilized by these kits^20^ and the elution of the extracted DNA in a large volume of buffer (usually between 50 and 150 µL). As a result, the extracted DNA will have low concentration, particularly in cases of lower bacteria concentration^20,21^. Additionally, they cannot completely remove inhibitory substances due to their lack of specificity for bacteria, exposure of magnetic silica beads (MSBs) to protein contamination during DNA extraction, and co-extraction of human DNA^15,20^. These factors reduce the sensitivity of molecular diagnostics by inhibiting DNA polymerase and by causing competition between non-target and target DNA^15,16^. Therefore, there is a need for immunomagnetic separation (IMS), for specific enrichment of bacteria from a large sample volume and the removal of inhibitory substances^22^. IMS provides a valuable solution to these challenges by enabling the specific capture and isolation of target bacteria from complex matrices, leading to improved sensitivity and specificity of molecular diagnostics for sepsis^22–24^.

However, while IMS offers many advantages, the materials employed in this method, such as silica-coated magnetic nanoparticles (SiO_2_-MNPs), come with their own set of limitations. Specifically, an oxide layer forms on their surfaces^25^, promoting non-specific interactions with cell membranes^26^. This undesirable interaction results in the inadvertent adsorption of blood components such as blood cells or platelets, leading to the aggregation of the SiO_2_-MNPs^27,28^. When these aggregated particles are used in subsequent steps, they can transfer inhibitory compounds during the DNA purification and extraction phase. This hinders the successful amplification of the target DNA during the polymerase chain reaction (PCR), potentially compromising the accuracy and reliability of diagnostic results. To circumvent this impediment, our research has pioneered an enhanced approach: coating MNPs with polydopamine (PDA) followed by conjugation with vancomycin (Van)^27^, which is known for its affinity to the peptidoglycan layer of Gram-positive bacteria^29,30^. Through this conjugation, selective enrichment of Gram-positive bacteria from blood samples can be achieved, effectively preventing aggregation. This achievement underscores a parallel need for: development of a similar strategy for selectively enriching Gram-negative bacteria.

In this study, we address the previously outlined challenges by introducing a novel method that conjugates allantoin (AL) to polydopamine-coated magnetic nanoparticles (PDA-MNPs). Notably, AL exhibits a specific affinity for lipopolysaccharide (LPS) on the outer membrane of Gram-negative bacteria^31^. Paired with vancomycin-conjugated PDA-MNPs (Van-PDA-MNPs), our innovative approach enables the preconcentration of an expansive spectrum of bacterial species. Further enhancing this methodology, we incorporated an automated magnet-based particle handling system, dubbed the automated magnetic separation system (AMSS). A remarkable finding of our research was that these nanoparticles enriched both Gram-positive and Gram-negative bacteria by more than 45-fold, achieving an impressive capturing efficiency exceeding 75% in blood samples. By employing qPCR, we were able to discern detection limits as low as 10^2^ CFU/mL across a range of bacterial species in both spiked blood and patient samples, a level of sensitivity that outperforms commercial kits by up to three orders of magnitude.

## Materials and methods

### Reagents

Iron (II, III) oxide, dopamine hydrochloride, allantoin, vancomycin hydrochloride, and oxacillin sodium salt were procured from Sigma-Aldrich (St. Louis, MO, USA). Phosphate-buffered saline (PBS, pH 7.4) was obtained from Gibco (Grand Island, NY, USA).

### Bacterial culture

The bacterial strains used in this study (Table 1) were obtained from American Type Culture Collection (ATCC, Manassas, VA, USA), Culture Collection of Antimicrobial Resistance Microbes (CCARM, Seoul, Korea) and Korean Collection for Type Culture (KCTC, Daejeon, Korea). For each bacterial strain, a single colony was transferred from the agar plate to 5 mL of the corresponding growth broth (LB, TSB, and BHIB), and the culture was incubated overnight at 37 °C and 200 rpm. The overnight culture was then diluted in fresh media and incubated under the same conditions to an optical density (OD) of 1 at 600 nm.

**Table 1:** Bacterial species and corresponding growth media.

Bacteria	Strain	Growth media
Escherichia coli O157:H7	ATCC 43,894	LB
Salmonella enteritidis	ATCC 13,076	LB
Pseudomonas aeruginosa	ATCC 39,327	LB
Klebsiella pneumoniae	ATCC 70,063	LB
Staphylococcus aureus	ATCC 27,213	TSB
Bacillus cereus	ATCC 21,722	LB
Staphylococcus epidermidis	KCTC 1917	TSB
Enterococcus faecalis	KCTC 3206	BHIB
Methicillin-resistant Staphylococcus aureus	CCARM 3107	TSB + oxacillin (6 μg/mL)

### Preparation of polydopamine coated magnetic nanoparticles conjugated with either vancomycin or allantoin

PDA-MNPs were synthesized by incubating 50 mg of iron oxide nanoparticles in 25 mL of a dopamine hydrochloride solution (2 mg/mL, pH 8.5)^27^. The mixture was continuously stirred for 3 h at room temperature (RT) to form a PDA coating on the surface of the MNPs. The PDA-MNPs were then separated using a permanent magnet and washed three times with phosphate-buffered saline (PBS, pH of 7.5).

To conjugate Van or Al to the surface of the PDA-MNPs, 25 mL of the respective solution (2 mg/mL, pH of 7.4) was added to the PDA-MNPs and incubated at RT for 3 h with vancomycin (Fig. 2a) or 12 h with allantoin (Fig. 2b). The resulting Van-PDA-MNPs and Al- PDA-MNPs were then separated using a permanent magnet washed three times with PBS, and suspended in 25 mL of PBS for further use. The size, morphology, and magnetic properties of the Van-PDA-MNPs and Al-PDA-MNPs were characterized before use.

### Transmission electron microscopy (TEM) imaging of MNPs

TEM images of MNPs, PDA-MNPs, Al-PDA-MNPs and Van-PDA-MNPs were acquired through the following procedure. Initially, MNPs underwent three washes with 1 mL of deionized (DI) water and were subsequently suspended in 1 mL of DI water. Following this, 10 µl of the particle suspension was dispensed onto a 300-mesh copper grid (CF-2/1-3CU-50 grid, Electron Microscopy Sciences, Hatfield, PA, USA) and allowed to dry in an oven at 70 °C for 2 h. Ultimately, the morphology and elemental map of the Al-PDA-MNPs and Van-PDA-MNPs were examined using a JEM-2100F transmission electron microscope (JEOL Ltd., Tokyo, Japan) operating at an accelerating voltage of 200 kV. The TEM was equipped with energy-dispersive X-ray spectroscopy (EDS) for elemental mapping.

### Zeta potential measurement

To assess the zeta potential of MNPs, PDA-MNPs, Al-PDA-MNPs, and Van-PDA-MNPs, a suspension of 0.2 mg of the MNPs was prepared in 1 mL of deionized (DI) water and analysed using a dynamic light scattering (DLS) instrument, Zetasizer Nano ZS (Malvern Instruments, Malvern, UK).

### Scanning electron microscopy (SEM) imaging of bacteria captured by Al-PDA-MNPs and Van-PDA-MNPs

For SEM imaging, bacterial suspensions (10^5^ CFU/mL) were mixed with either Van-PDA-MNPs or Al-PDA-MNPs (10^11^ particles/mL). Following the mixture, unattached bacteria were removed by washing twice with PBS. The resulting bacterial-MNP mixtures were fixed in 2% glutaraldehyde for 1 h^27^, rinsed three times with PBS, treated with 1% osmium tetroxide (1 h, 4 °C, dark), and dehydrated using graded ethanol. Next, 10 μL of the resulting complex was deposited on a 200-mesh copper grid and air-dried for 2 h. SEM imaging was conducted using a JSM7000F SEM (JEOL Ltd.), at accelerating voltage of 5 kV and a magnification of 15,000× .

### Manual preconcentration of bacteria in PBS and blood

One milliliter of either PBS or blood, containing bacterial concentrations ranging from 10^1^ to 10^4^ CFU/mL, was mixed with 200 µL of Van-PDA-MNPs or Al-PDA-MNPs at a concentration of 10^11^ particles/mL. This mixture was incubated at 37 °C for 20 min, in line with the protocols detailed in our previous publications^27,28^. Subsequently, the resulting bacteria-MNP complexes were isolated using a magnetic separation rack (Bioneer Co. Daejeon, Korea), and the eluent was collected and plated on LB agar plates for standard colony counting. To determine the capture efficiency of the MNPs for each bacterial species, we used the following equation^32^:

$$Capturing \,efficency \,\left(\%\right)=\left(\frac{{N}_{t}-{N}_{u}-{N}_{p} }{{N}_{t}}\right)100$$

where ${N}_{t}$ is the total number of bacterial cells present in the sample, and ${N}_{u}$ is the number of bacterial cells remaining unbound after treatment with Van/Al-PDA-MNPs, and *Np* is the number of bacterial cells captured non-specifically by non-functionalized PDA-MNPs.

The preconcentration fold was determined using the following equation^33^.

$$Preconcentration \,fold=Capturing \,efficiency*(\frac{Initial \,volume}{Preconcentrated \,volume})$$

### Automated Magnetic Separation System (AMSS)

**Figure 1:**
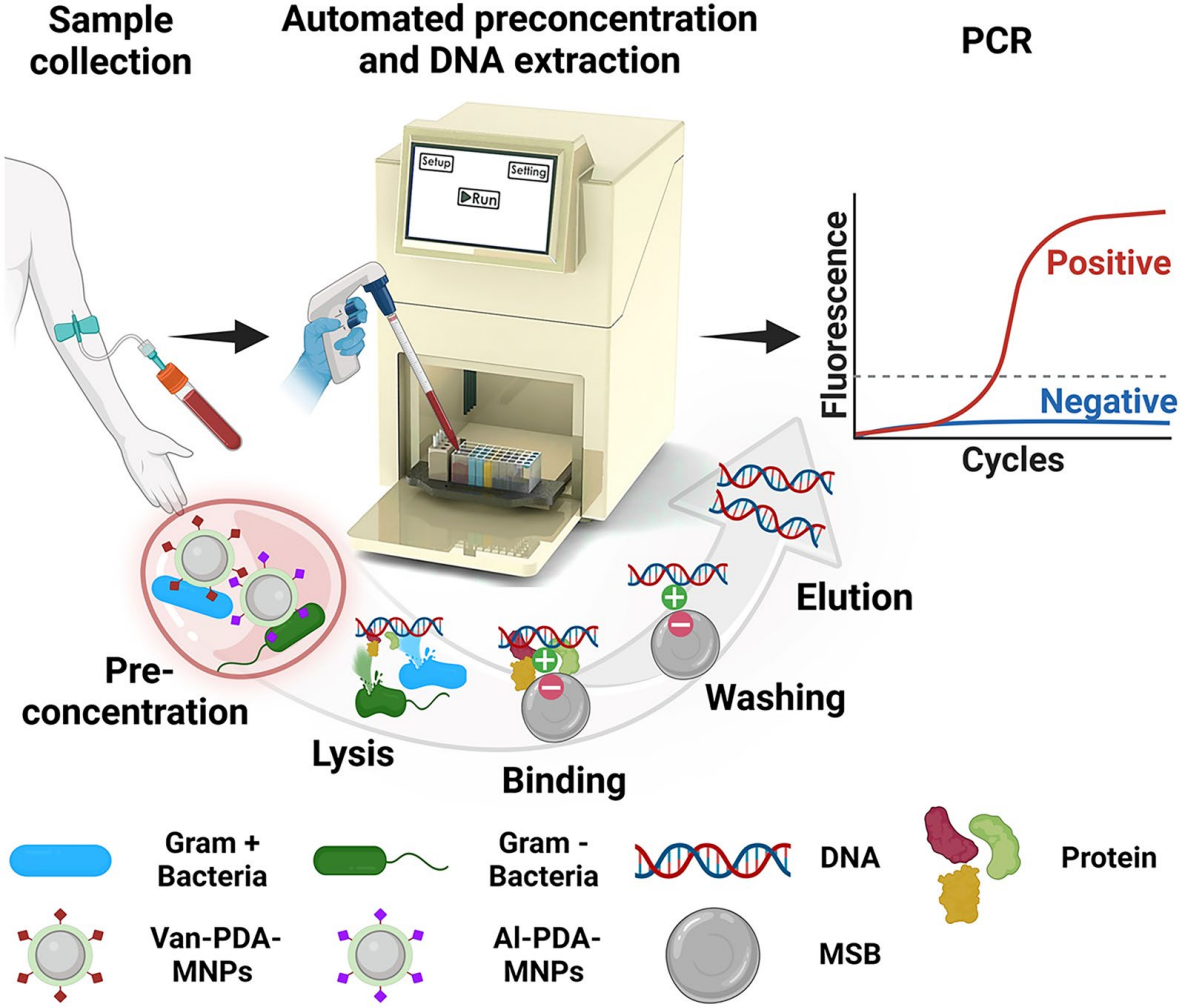
Schematic representation of the setup for bacterial preconcentration and DNA extraction, utilizing vancomycin (Van) and allantoin (AL)-conjugated polydopamine (PDA)-coated magnetic nanoparticles (MNPs), geared towards an advanced automated diagnostic system for sepsis. MSB stands for magnetic silica beads.

Figure 1 outlines the methodology of the automated magnetic separation system and its integration with PDA-coated MNPs. The compact system, measuring 320 × 235 × 360 mm, features a magnetic rod and a plastic tip holder, both controlled by a stepper motor for vertical and horizontal motions (Figure S1). The well plate is structured with four lines, each comprising nine wells, facilitating the simultaneous processing of up to four samples. The first three wells are designated for bacteria preconcentration. The initial well, with a capacity of 2.5 mL of blood, and the subsequent two each, with a capacity of 1 mL, are designated for washing the bacteria-MNP complex with PBS. The subsequent six wells are dedicated to DNA extraction steps: bacterial lysis, DNA binding to magnetic silica beads, three washing stages, drying, and elution. Buffer capacities vary, with the initial four wells in this section holding up to 1 mL, and the final two accommodating 0.4 mL.

### Bacterial concentration and DNA separation from bacteria in spiked blood and patient samples using AMSS

Blood with 0.1% K2 EDTA was obtained from Innovative Research Inc. (Novi, MI, USA) and its use was approved by the Institutional Review Board (IRB) of SKKU (approval no. SKKU 2017-11-006). This blood was spiked with nine bacterial species at concentrations ranging from 10^1^ to 10^4^ CFU/mL. Subsequently, 2.5 mL of these samples were mixed with both Al-PDA-MNPs and Van-PDA-MNPs in well 1 for a duration of 20 min. Using magnetic bars equipped with plastic tips, the MNP-bacteria complexes were collected, washed twice in well 2 and 3 respectively, and transferred to well 4 for bacterial lysis. Following lysis, the MNPs were discarded to well 3, and DNA was captured on MSBs (400 nm; MagListo™ 5 M Genomic DNA extraction kit, Bioneer, Korea) in well 4. These beads were cleaned in three wash steps in well 5, 6 and 7 and finally moved to an elution buffer well to release the DNA for subsequent PCR analysis.

Patient serum samples, each comprising 500 µL and provided by Asan Medical Centre, underwent a similar procedure for bacteria preconcentration and DNA extraction. The Institutional Ethics Committee of Asan Medical Center granted an exemption for this study, aligning with the Bioethics and Safety Act (IRB exemption confirmation: 2022–0914). All experiments were performed in accordance with the relevant guidelines and regulations. Informed consent was obtained from all subjects and/or their legal guardian(s).

#### PCR and qPCR conditions and primer information for gene amplification

Two thermal cyclers were utilized for PCR and qPCR: the MJ MINI thermocycler (Bio-RAD, Hercules, CA, USA) for PCR and the StepOne™ real-time PCR system (Applied Biosystems, Foster City, CA, USA) for qPCR. The qPCR protocol comprised 45 cycles, including denaturation at 95 °C for 15 s, annealing at 56 °C for 30 s, and extension at 72 °C for 30 s. For PCR, the protocol included initial denaturation at 95 °C for 5 min, followed by 40 cycles of denaturation at 95 °C for 15 s, annealing at 56 °C for 30 s, and extension at 72 °C for 30 s. A final extension at 72 °C for 5 min. PCR products were separated on a 2% TAE agarose gel, running at 100 V for 30 min. The primer sequences employed for both PCR and qPCR are listed in Table 2.

**Table 2:** Nucleotide sequences of primers for PCR and qPCR amplification.

Bacteria (gene, amplicon size)	Primer orientation	Sequence (5′-3′)	Reference
Universal (16 s rRNA, 466 bp)	F	TCCTACGGGAGGCAGCAGT	^35^
	R	GGACTACCAGGGTATCTAATCCTGTT	
*E. coli* (eaeA, 150 bp)	F	GGCGGATTAGACTTCGGCTA	^36^
	R	CGTTTTGGCACTATTTGCCC	
*S. enteritidis* (invA, 115 bp)	F	AGCGTACTGGAAAGGGAAAG	^37^
	R	ATACCGCCAATAAAGTTCACAAAG	
*P. aeruginosa* (gyrB, 222 bp)	F	CCTGACCATCCGTCGCCACAAC	^38^
	R	CGCAGCAGGATGCCGACGCC	
*K. pneumoniae* (rcsA, 176)	F	GGATATCTGACCAGTCGG	^39^
	R	GGGTTTTGCGTAATGATCTG	
*S. aureus* (nuc, 207 bp)	F	ACACCTGAAACAAAGCATCC	^40^
	R	TAGCCAAGCCTTGACGAACT	
*B. cereus* (gyrB, 220 bp)	F	GCCCTGGTATGTATATTGGATCTAC	^41^
	R	GGTCATAATAACTTCTACAGCAGGA	
MRSA(mecA, 134 bp)	F	AACCACCCAATTTGTCTGCC	^42^
	R	TGATGGTATGCAACAAGTCGTAAA	
*S. epidermidis* (gseA, 194 bp)	F	GGCAAATTTGTGGGTCAAGA	^43^
	R	TGGCTAATGGTTTGTCACCA	
*E. faecalis* (ddl, 84 bp)	F	GACAGGAAAGAAACTAGGAGGAC	^44^
	R	AAACAGACACATCGTGCT	

#### Statistical analysis

The reported results represent the mean ± standard deviation of three independent experiments. Statistical analysis was conducted using Student’s t-test to compare the data acquired under different conditions. Results with a *p*-value less than 0.05 were considered significant. Notations for significance levels is as follows: *: *p* < 0.05. **: *p* < 0.01. ***: *p* < 0.001.

## Results and discussion

### Characterization of MNPs, PDA-MNPs, Van-PDA-MNPs and Al-PDA-MNPs

**Figure 2:**
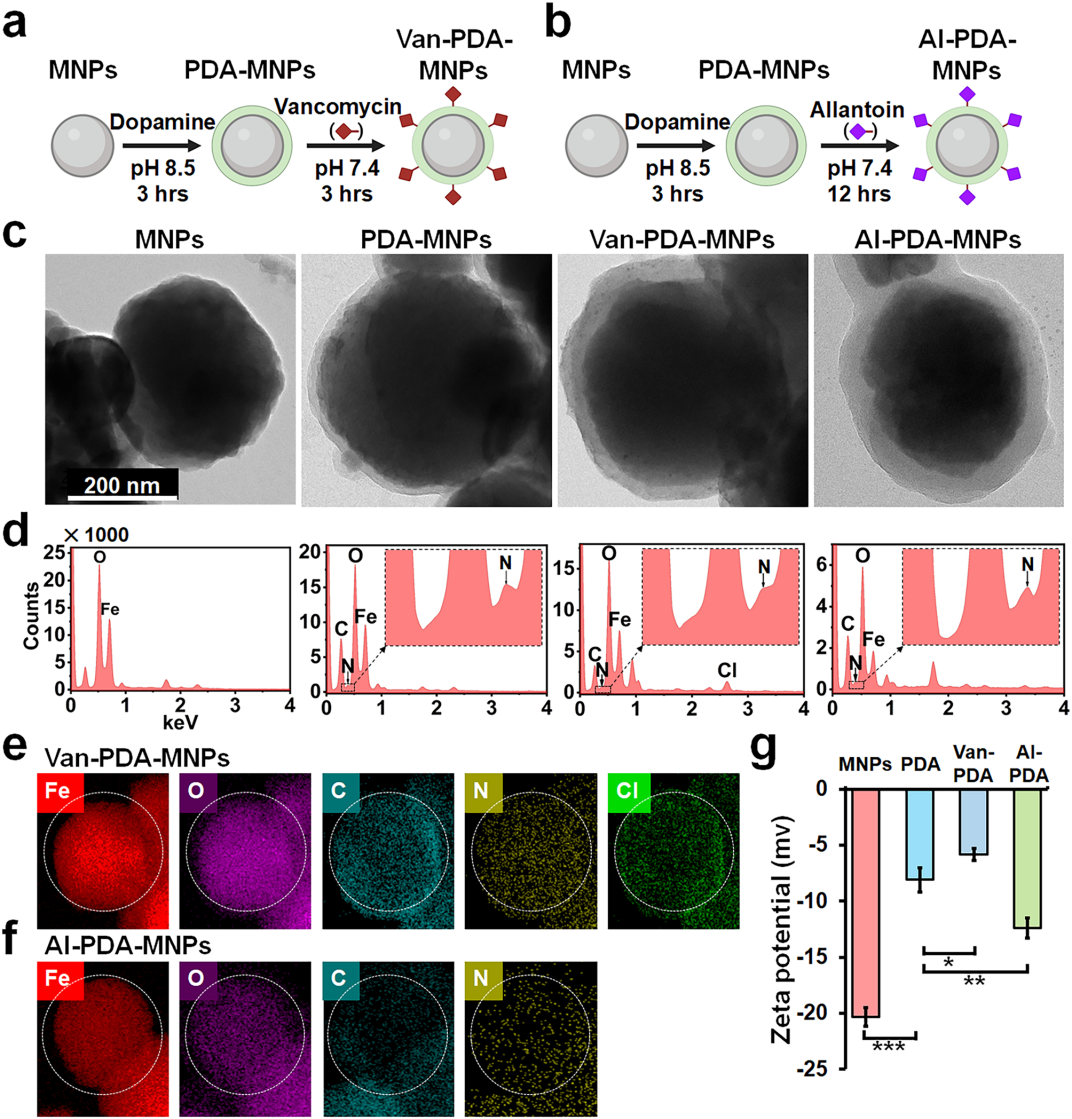
Synthesis and characterization of MNPs. (**a**, **b**) Schematic illustrations of the synthetic process for (**a**) Van-PDA-MNPs and (**b**) Al-PDA-MNPs. (**c**) Transmission electron microscopy (TEM) images of MNPs (MNPs, PDA-MNPs, Van-PDA-MNPs and Al-PDA-MNPs. Energy-dispersive X-ray spectroscopy (EDS) elemental analysis (**d**) of all MNPs and elemental mapping (**e**, **f**) of Van-PDA-MNPs and Al-PDA-MNPs. (**g**) Zeta potential measurements of MNPs (MNPs, PDA-MNPs, Van-PDA-MNPs, and Al-PDA-MNPs) by dynamic light scattering (DLS) using the Zetasizer Nano ZS instrument (Malvern Instruments, Malvern, UK). Student’s t-test, ***: P < 0.001. **: P < 0.01.*: P < 0.05. n = 3.

The synthesis of Van-PDA-MNPs and Al-PDA-MNPs began by applying a PDA layer to bare MNPs. This step was followed by the grafting of either Van) or Allantoin (Al) onto the PDA-coated MNPs. The grafting mechanism utilized the reaction between the primary amino group in Van or the urea component in Al with the aromatic rings present in PDA. This procedure is illustrated in Fig. 2a and b. TEM imaging revealed that the iron oxide nanoparticles mainly possess a dark core. When coated with PDA, a clear layer surrounds this core, as seen in Fig. 2c. While the dark core represents iron oxide, the clear layer signals the presence of PDA. However, distinguishing between solely PDA-coated MNPs and those further conjugated with Van or Al using only TEM is challenging.

Hence, we used EDS analyses to validate the presence of PDA, Al, and Van on Al-PDA-MNPs and Van-PDA-MNPs surfaces (Fig. 2d). EDS results for the uncoated MNPs highlighted peaks for Fe and O, suggesting an iron oxide core. The N peak and enhanced C and N peaks confirmed the presence of PDA. For Van-PDA-MNPs, a unique Cl peak denoted successful grafting of vancomycin onto PDA. Since allantoin's elements overlap with PDA, discerning specific peaks for it was not possible in the EDS. EDS mapping (Fig. 2e, f) showcased the distribution of elements including Fe, O, C, and N across Al-PDA-MNPs and Van-PDA-MNPs, reinforcing the TEM findings and affirming the iron oxide nanoparticle core and PDA coating composition.

The surface charge of these MNPs was analyzed to understand the impact of the PDA coating and the subsequent conjugations (Fig. 2g). Uncoated MNPs exhibited a charge of − 20.33 ± 0.82 mV. After PDA coating, the MNPs had a reduced negative charge, registering at − 8.08 ± 1.09 mV. When further modified with Al-PDA and van-PDA, the MNPs displayed charges of − 12.40 ± 0.93 mV and − 5.81 ± 0.54 mV, respectively. These results highlight the significant influence of the PDA coating and conjugation on the MNPs' surface charge, suggesting successful coating and binding processes.

### Manual preconcentration of different species of bacteria with Al-PDA-MNPs and Van-PDA-MNPs in PBS and blood

**Figure 3:**
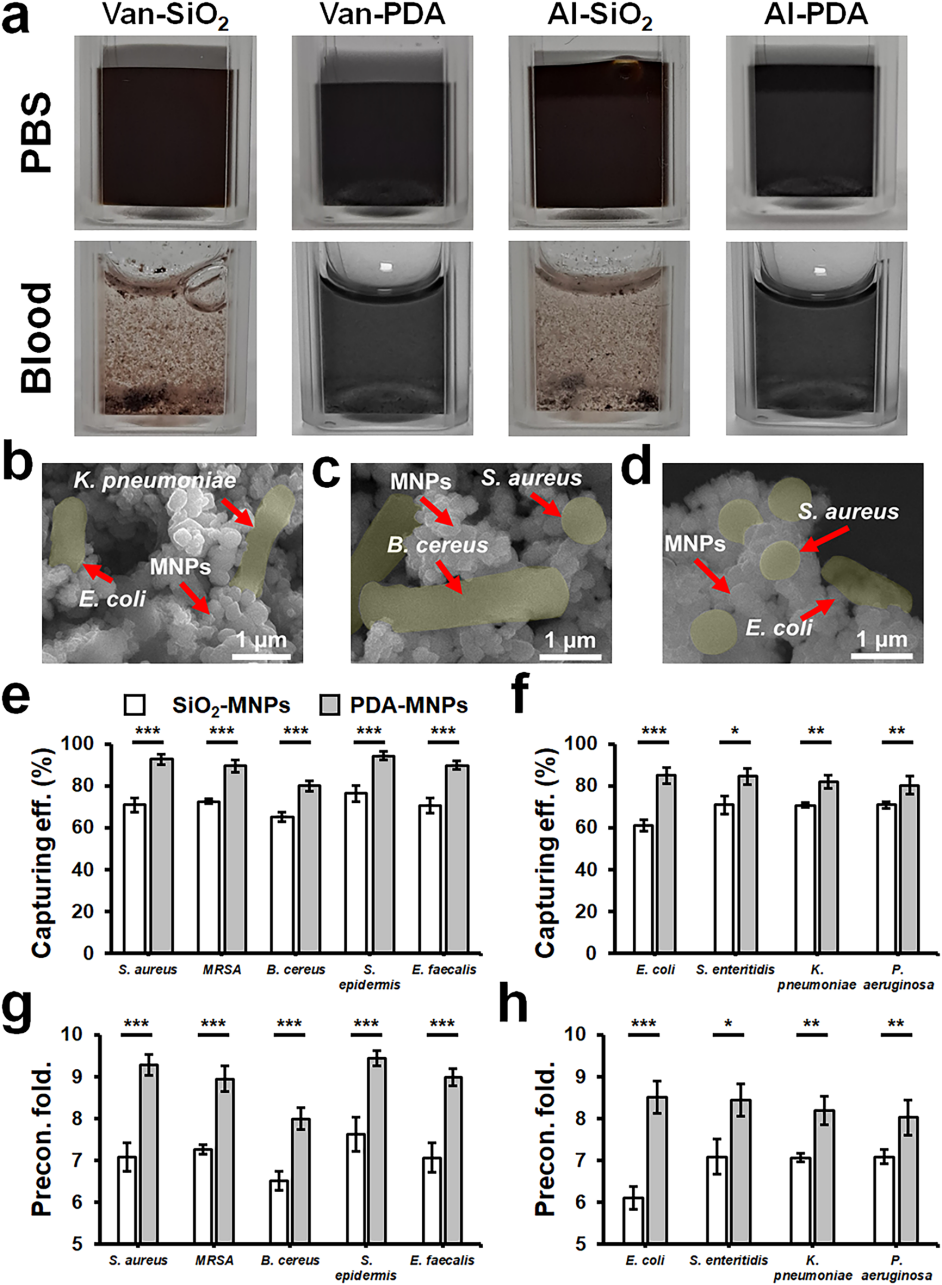
Interaction between MNPs, blood cells, and various bacterial strains**.** (**a**) Dispersion characteristics of particles in PBS and blood. (**b–d**) SEM images of bacteria captured by PDA-PDAs: (**b**) Gram-negative bacteria (*K. pneumoniae* and *E. coli*) captured by Al-PDA-MNPs; (**c**) Gram-positive bacteria (*S. aureus* and *B. cereus*) captured by Van-PDA-MNPs; (**d**) Capture of Gram-positive and Gram-negative strains using combined Van-PDA-MNPs and Al-PDA-MNPs. SEM images were captured employing a JSM7000F (JEOL Ltd.) scanning electron microscope, operated at a 5 kV accelerating voltage and set to a magnification level of 15,000× . (**e–h**) Effect of SiO_2_ and PDA coating on capturing efficiency and preconcentration fold of Gram-positive and Gram-negative bacteria in PBS by the MNPs conjugated with Van or AL: capturing efficiency of Gram-positive (**e**) and Gram-negative bacteria (**f**); Preconcentration fold of Gram-positive (**g**) and Gram-negative (**h**) bacteria; all experiments on capturing efficiency and preconcentration fold were carried out with a bacteria concentration of 10^4^ CFU/mL and a particle concentration of 10^11^ particles/mL (final conc.) with a 20-min incubation at 37 °C. Magnetic separation of MNPs was performed using MagListo™ magnetic separation rack (Bioneer, Daejeon, Korea). Student’s t-test, ***: P < 0.001. **: P < 0.01.*: P < 0.05. n = 3.

Preliminary experiments with *S. aureus*, across concentrations ranging from 10^1^ to 10^4^ CFU/mL, showed a decrease in capturing efficiency with increasing bacterial concentration. For a detailed evaluation of capturing efficiency under diverse conditions, we selected a concentration of 10^4^ CFU/mL (Fig. S2). Subsequent experiments were conducted to investigate the polydopamine coating's effectiveness in preventing MNPs aggregation in blood and to compare its performance with conventionally used silica-coated MNPs. In PBS, no observable aggregation was detected among the magnetic nanoparticles (MNPs) investigated, which included conventionally used silica-coated MNPs conjugated with Van and Al (Van-SiO_2_-MNPs and Al-SiO_2_-MNPs), as well as PDA coated MNPs (Van-PDA-MNPs and Al-PDA-MNPs). Conversely, in blood, silica-coated MNPs aggregated, exhibited aggregation, a phenomenon conspicuously absent in the case of Van-PDA-MNPs and Al-PDA-MNPs (Fig. 3a). This corroborates our earlier findings that PDA coatings prevents aggregation with vancomycin-conjugated MNPs^27^. These results underscore the superiority of PDA coatings in preventing unwanted MNP clustering, preserving their effectiveness in bacterial preconcentration.

SEM was employed to confirm the successful capture of bacteria by Al-PDA-MNPs and Van-PDA-MNPs across various Gram-positive and Gram-negative bacterial strains (Fig. 3b–d). The SEM images show bacteria bound to the MNP surface. Intriguingly, SEM also demonstrated concurrent capture of diverse Gram strains using a mix of MNPs, as evidenced by *E. coli* and *S. aureus* co-capture in one sample (Fig. 3d). Such images underscore the prowess of PDA-coated MNPs combined with Van and Al in isolating and concentrating multiple bacterial targets. Further testament to this effectiveness was observed in colony counting visuals, showcasing successful immunomagnetic separations with Van-PDA-MNPs and Al-PDA-MNPs in an automated setup (Figure S3). Together these findings emphasize the potential of the technique to revolutionize diagnostic processes by preconcentrating a variety of bacterial species.

A comparison of capturing efficiency between conventionally used silica-coated magnetic nanoparticles (MNPs) and Polydopamine (PDA)-coated MNPs in phosphate-buffered saline (PBS) is depicted in Fig. 3e, f. This comparison underscores the superiority of PDA-coated MNPs over their conventional counterparts, leading to higher preconcentration efficiency, as evidenced in Fig. 3g, h. Notably, even in a PBS environment, PDA-coated MNPs surpass the performance of their SiO_2_-coated counterparts. This heightened efficacy, as suggested by Zhao et al*.,* is due to the increased surface area offered by PDA coatings, which present an abundance of amino and hydroxyl group binding sites^34^. This increased surface area of PDA-coated MNPs is presumed to result in a more concentrated presence of vancomycin on PDA-MNPs, enhancing their bacterial capture potential. On the other hand, SiO_2_-coated MNPs fall short, particularly in blood samples, due to their tendency for aggregation.

### Automated preconcentration of different species of bacteria with Al-PDA-MNPs and Van-PDA-MNPs in PBS and blood

**Figure 4:**
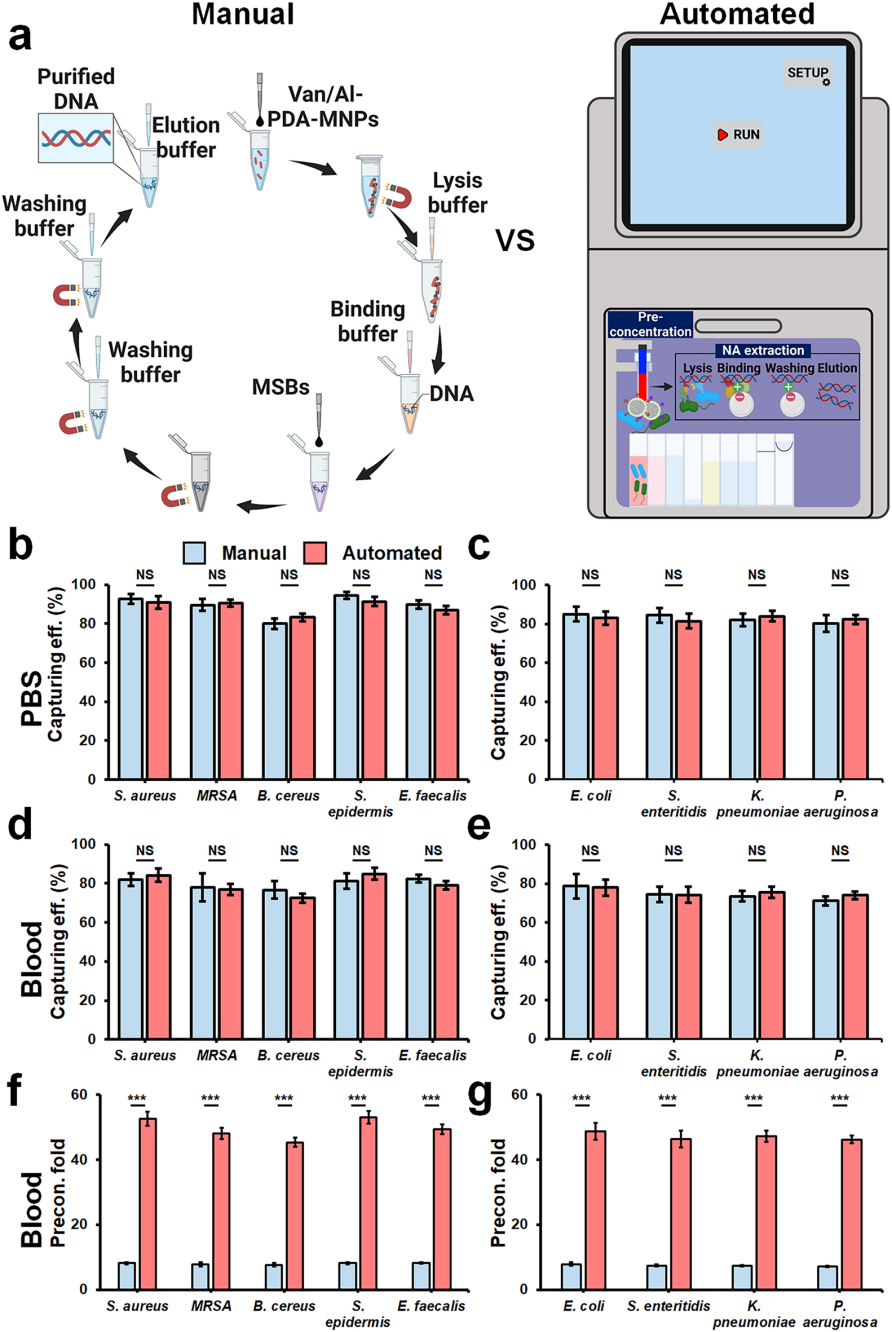
Comparative analysis of manual vs. automated magnetic separation system (AMSS) for bacterial capturing using PDA-MNPs. (**a**) Schematic showcasing the distinctions between manual and AMSS sample preparation approaches. (**b**, **c**) Efficiency comparison in PBS between manual and AMSS using Van-PDA-MNPs at a bacterial concentration of 10^4^ CFU/mL. (**d**, **e**) Efficiency assessment of both methods using Al-PDA-MNPs at the same concentration. (**f**, **g**) Preconcentration fold evaluation for both manual and AMSS in blood with Van-PDA-MNPs and Al-PDA-MNPs. For the procedures, 1 mL of either PBS or blood with bacteria at 10^4^ CFU/mL was combined with 200 µL of Van-PDA-MNPs or Al-PDA-MNPs at a concentration of 10^11^ particles/mL. After incubation at 37 °C for 20 min, the resultant bacteria-MNP complexes were separated using either the MagListo™ magnetic separation rack or the AMSS. The eluent was then plated on the corresponding agar plates, and colonies were counted using standard methods.

Comparison between automated and manual preconcentration setups using Van-PDA-MNPs and Al-PDA-MNPs (Fig. 4a) in PBS and blood revealed similar capture efficiencies. Automated preconcentration demonstrated comparable performance to the manual method for both Van-PDA-MNPs and Al-PDA-MNPs in this study. (Figs. 4b–e). In PBS, Van-PDA-MNPs captured Gram-positive bacteria such as *S. aureus*, methicillin-resistant *S. aureus* (MRSA), and others with efficiencies exceeding 80% (Fig. 4b), while the efficiency in blood remained above 70% (Fig. 4d). Conversely, Al-PDA-MNPs captured Gram-negative bacteria like *E. coli* and *S. enteritidis* with efficiencies around 80% in PBS (Fig. 4c) and about 70% in blood (Fig. 4e).

In exploring the efficacy of Van-PDA-MNPs, we extended our investigation to include vancomycin-resistant species, particularly vancomycin-resistant Enterococcus (VRE). Our data revealed that the Van-PDA-MNPs achieved a capturing efficiency of 88% in PBS and 81% in whole blood samples for VRE. These results are comparable to those obtained with non-resistant bacterial strains, suggesting that the nanoparticles' capturing mechanism is not impeded by the resistance factor. This finding is particularly encouraging as it demonstrates the potential of Van-PDA-MNPs to effectively capture a broad spectrum of bacteria, regardless of their resistance to vancomycin, which is pivotal for their application in diverse clinical scenarios (refer to Figure S4 for graphical data).

The remarkable capturing efficiencies observed in this study are attributed to the distinctive properties of the magnetic nanoparticles. The polydopamine coating plays a crucial role in minimizing non-specific adsorption of blood cells and platelets. Furthermore, the Van-PDA-MNP exhibits notable capabilities in capturing various Gram-positive bacteria, primarily owing to the high affinity of vancomycin for the peptidoglycan layer. The Al-PDA-MNP demonstrates superior efficiency in capturing Gram-negative bacteria, attributed to the presence of amide groups on the allantoin surface. These amide groups form bonds with the lipopolysaccharides of the bacteria^31^. As a result, these MNPs are promising tools for simultaneous capture and enrichment of diverse bacterial species, which may prove invaluable for applications like disease diagnosis and treatment.

The specially designed automated system, with the ability to process sample volumes up to 2.5 mL, demonstrated outstanding preconcentration efficiency. This resulted in a significant enhancement in preconcentration fold (Fig. 4f, g**)**. This underscores the potential of PDA-coated MNPs to efficiently handle large-volume blood samples in an automated setting.

### Enhancing Sepsis Molecular Diagnostics through Automated Preconcentration and DNA Extraction in Spiked Blood and Clinical Samples

**Figure 5:**
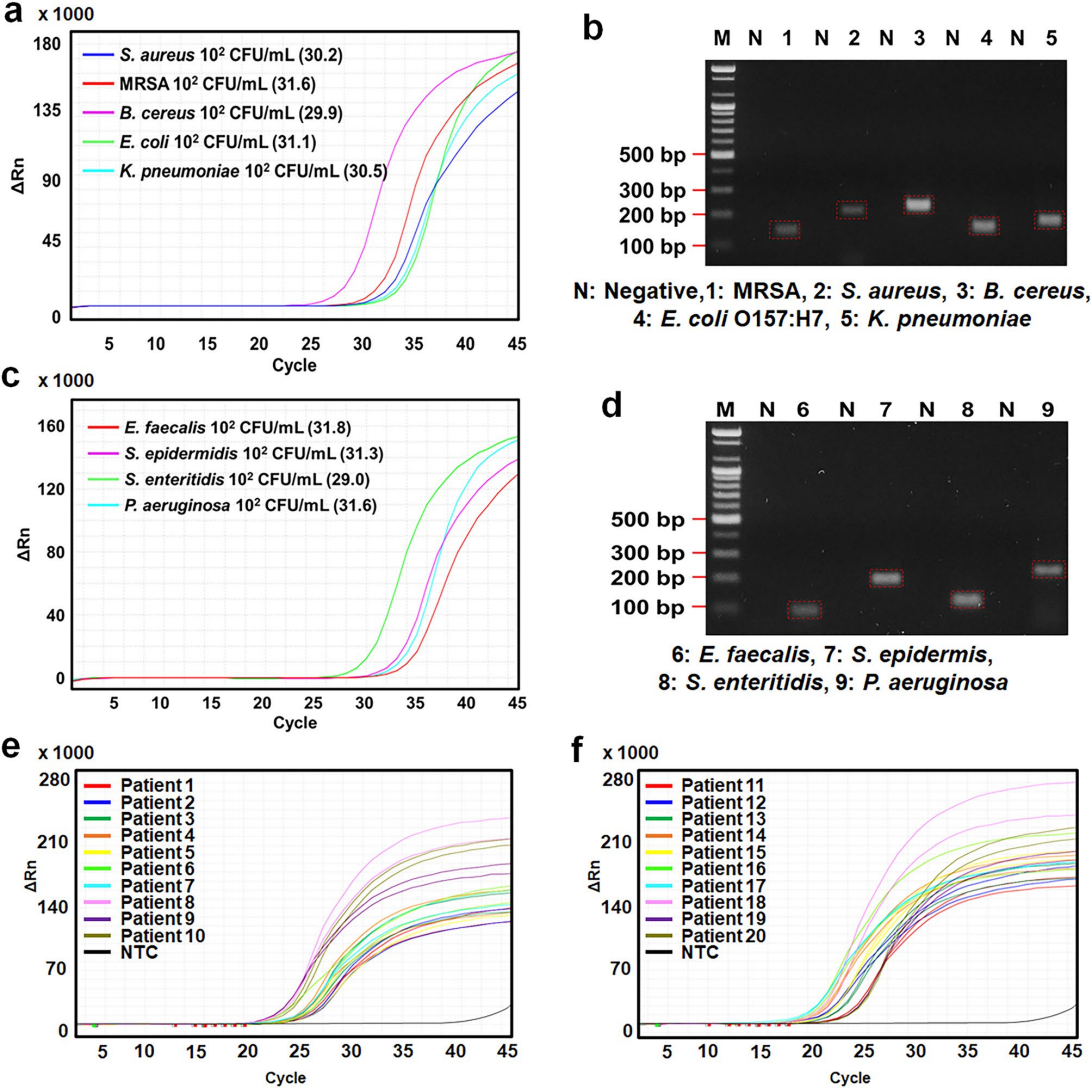
Results of qPCR and gel electrophoresis. (**a**, **c**) qPCR curves showcasing the DNA samples of 9 bacterial species after preconcentration of bacteria and DNA extraction from blood via the automated sample preparation system. (**b**, **d**) Gel electrophoresis images corresponding to the DNA samples mentioned above. (**e**, **f**) qPCR analyses targeting the amplification of the 16S rRNA gene to identify the presence of bacteria in patient samples.

After the successful integration of our automated system for preconcentration and DNA extraction, we conducted an in-depth analysis using quantitative polymerase chain reaction (qPCR). We aimed to identify target bacteria in both spiked blood samples and actual patient samples sourced from Asan Medical Centre. Based on the results shown in Figs. 5a–d, our methodology demonstrates high effectiveness. The qPCR curves, paired with the gel electrophoresis images, confirm our capability to detect bacterial concentrations as low as 10^2^ CFU/mL for both Gram-positive and Gram-negative strains. This impressive sensitivity is attributed to the use of polydopamine (PDA)-coated nanoparticles in the initial automated preconcentration phase, further enhanced by DNA extraction from 2.5 mL of blood.

Figures 5e and f provide a detailed overview of our microbial identification approach. We utilize 16S ribosomal RNA (rRNA) amplification to detect the presence of bacteria in patient samples. Once a sample is confirmed positive, species-specific primers are employed to refine our identification process. This additional step allows us to accurately identify the specific bacteria causing the infections. The outcomes of our method, which enable precise bacterial identification, are documented in Tables 3 and 4, showcasing the specific bacteria detected in each patient's sample.

**Table 3:** Species of Gram-negative bacteria detected in the patient samples.

Patient	*S. enteritidis*	*E. coli*	*K. pneumoniae*	*P. aeruginosa*
I176	** + **	**–**	**–**	**–**
I465	**–**	**–**	** + **	** + **
I554	**–**	** + **	** + **	** + **
E80	** + **	**–**	**–**	**–**
E96	** + **	**–**	**–**	** + **
E850	**–**	** + **	**–**	**–**
E887	**–**	** + **	**–**	**–**
E926	**–**	** + **	**–**	**–**
E986	**–**	**–**	**–**	** + **
E1389	**–**	** + **	**–**	**–**

**Table 4:** Species of Gram-positive bacteria detected in the patient sample.

Patient	*S. aureus*	*MRSA*	*B. cereus*	*S. epidermidis*	*E. faecalis*
E4	–	+	–	–	–
E25	+	+	–	–	–
E209	+	+	–	–	–
E861	–	–	–	–	+
E935	–	–	–	–	+
I290	+	–	+	–	–
I293	+	–	–	–	–
I338	–	–	–	–	–
I444	–	–	–	+	–
I848	+	–	–	–	+

In essence, our data from actual patient samples highlight the efficacy of our automated platform's preconcentration and DNA extraction procedures. The broader significance of our work is profound; our innovative approach holds promise for rapid and precise detection of sepsis-inducing bacteria in blood samples, heralding advancements in clinical diagnostics and ultimately enhancing patient outcomes.

## Conclusion

This study highlights the remarkable capabilities of Van- and Al-conjugated PDA-coated MNPs in rapidly and automatically preconcentrating bacteria and facilitating their DNA extraction. The strategic application of a PDA coating effectively addresses the problem of MNP aggregation, enabling the handling of larger sample volumes. This enhances both the detection limit and specificity of the process. A significant advantage of the IMS method is its ability to bypass initial sample pretreatment steps, streamlining bacterial preconcentration and DNA extraction, and thus presenting an efficient approach in these processes.

This method stands out in clinical settings due to its exceptional rapidity, affordability, and capacity for processing large sample volumes. The entire sample preparation process can be expedited to under one hour, significantly enhancing sensitivity and facilitating quicker sepsis diagnosis. Such efficiency and speed enable the method's seamless integration into healthcare facilities of varying sizes, from local clinics to major hospitals, thereby improving patient outcomes through timely intervention. Furthermore, the magnetic nanoparticles (MNPs) utilized are economically viable, with the cost per test being a mere 0.35 USD. This affordability is a pivotal advantage, promoting the method's potential for widespread clinical adoption and positioning it as a cost-effective solution in the global fight against sepsis.

It is essential to acknowledge that the present system's detection capability is limited to 100 CFU/mL from a 2.5 mL blood sample across all nine bacterial types tested. Addressing lower bacterial concentrations, particularly levels as minimal as 1 CFU/mL, requires further refinement of the system. Our current endeavors are focused on optimizing the system for processing larger blood volumes, up to 10 mL, with the goal of lowering the detection threshold to 1 CFU/mL. Such an enhancement is critical for increasing the sensitivity of our diagnostic platform, thereby rendering it more applicable and effective in clinical settings where stringent detection capabilities are necessary. Additionally, incorporating PCR or isothermal amplification methods into the diagnostic platform presents the opportunity for full automation, which could eliminate the necessity for manual intervention. This advancement would streamline the diagnostic procedure, enhancing both the efficiency and reliability of the system. Such a development positions the platform as a more comprehensive and automated solution for sepsis diagnostics, potentially transforming its application in medical facilities.

### Supplementary Information

Supplementary Information.

## Data Availability

The data supporting this study are included in this published article and its Supplementary Information.
