# Author Correction: Automated sepsis detection with vancomycin- and allantoin-polydopamine magnetic nanoparticles

**DOI:** 10.1038/s41598-024-55791-2

**Published:** 2024-03-13

**Authors:** Abdurhaman Teyib Abafogi, Jinyeop Lee, Joochan Kim, Sei Won Lee, Seongsoo Jang, Sungsu Park

**Affiliations:** 1 School of Mechanical Engineering, Sungkyunkwan University, Suwon, 16419 Korea; 2 KingoBio Inc., Seoul, 08390 Korea; 3 Department of Pulmonology and Critical Care Medicine, Asan Medical Center, University of Ulsan College of Medicine, Seoul, 05505 Korea; 4 Department of Laboratory Medicine, Asan Medical Center, University of Ulsan College of Medicine, Seoul, 05505 Korea

Correction to: *Scientific Reports* 10.1038/s41598-024-54236-0, published online 14 February 2024

The original version of this Article contained an error, where 10^1^ was incorrectly written as 10^4^.

As a result, in the “Materials and Methods” section, under the subheading “Manual preconcentration of bacteria in PBS and blood”,

“One milliliter of either PBS or blood, containing bacterial concentrations ranging from 10^4^ to 10^4^ CFU/mL, was mixed with 200 μL of Van-PDA-MNPs or Al-PDA-MNPs at a concentration of 10^11^ particles/mL.”

now reads:

“One milliliter of either PBS or blood, containing bacterial concentrations ranging from 10^1^ to 10^4^ CFU/mL, was mixed with 200 μL of Van-PDA-MNPs or Al-PDA-MNPs at a concentration of 10^11^ particles/mL.”

The original Article has been corrected.

