# Supplementary material for: Automated sepsis detection with vancomycin- and allantoin-polydopamine magnetic nanoparticles
Source: Sci Rep. 2024 Feb 14;14:3693. doi: 10.1038/s41598-024-54236-0 (PMC10867076; doi:10.1038/s41598-024-54236-0)
Supplement: Supplementary file 1 — Supplementary Information. [file 41598_2024_54236_MOESM1_ESM.docx]

**Automated Sepsis Detection with Vancomycin- and Allantoin-Polydopamine Magnetic Nanoparticles**

Abafogi Abdurhaman Teyib^1^, Jinyeop Lee^1, 2^, Joochan Kim^1^, Sei Won Lee^3^, Seongsoo Jang^4^ & Sungsu Park^1^*

^1^School of Mechanical Engineering, Sungkyunkwan University, Suwon 16419, Korea

^2^ KingoBio Inc., Seoul, 08390, Korea

^3^Department of Pulmonology and Critical Care Medicine, Asan Medical Center, University of Ulsan College of Medicine, Seoul, 05505, Korea

^4^Department of Laboratory Medicine, Asan Medical Center, University of Ulsan College of Medicine, Seoul, 05505, Korea

***** Correspondence: nanopark@skku.edu


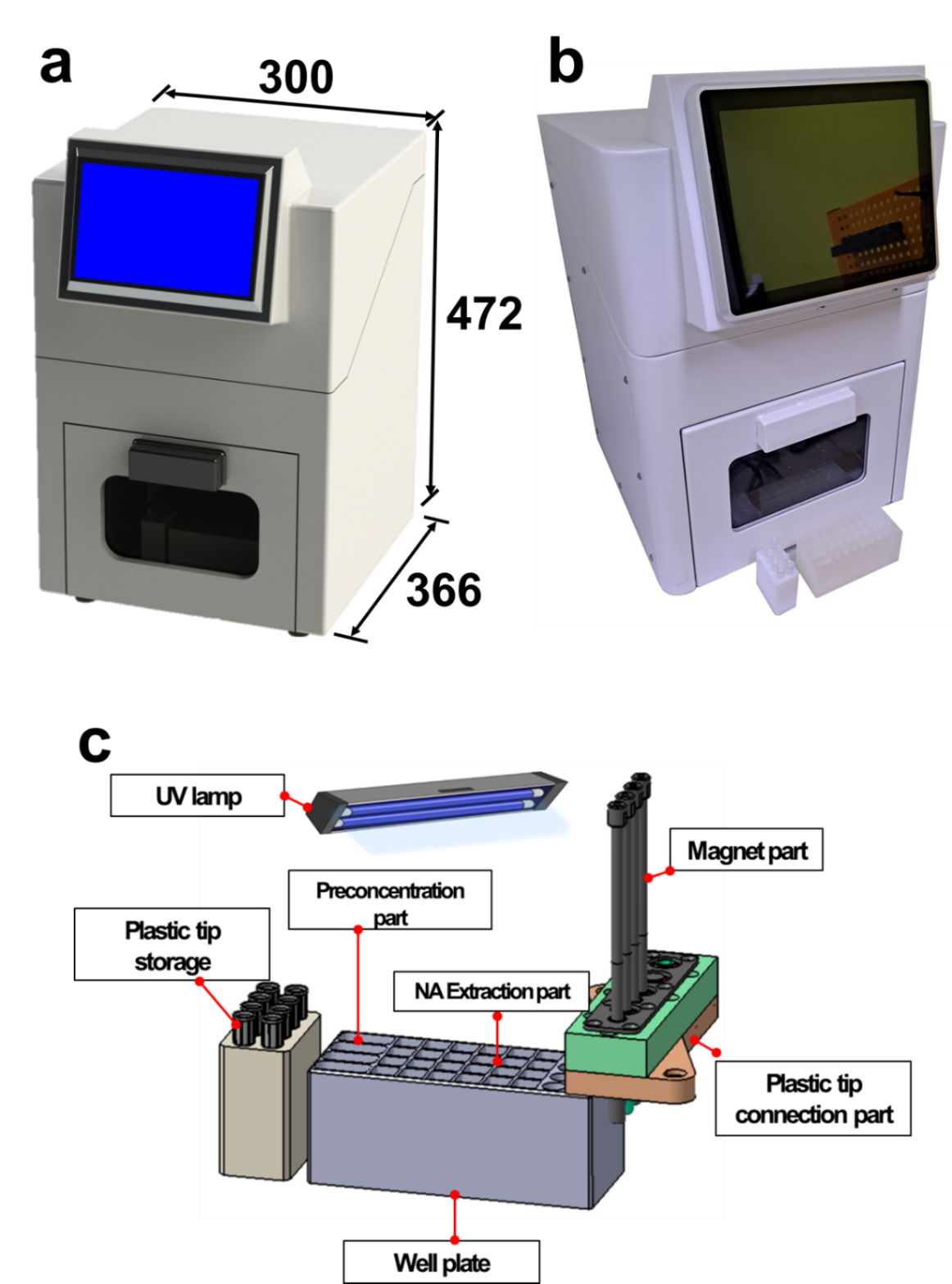


**Figure S1.** 3D model **(a)** and photographic image **(b)** of the automated system and its complementary components **(c)**. The well plate and plastic tip displayed in figure S1c were produced using Inventor® Professional Student Edition software from Autodesk Inc. located in Seoul, Korea. Both components were fabricated from poly (methyl methacrylate), commonly known as acrylic glass, using CNC machining. The well plate measures 124 × 120 × 47 mm (length × width × height), while the plastic tip has dimensions of 120 ×10 × 2 mm (length × diameter × thickness).


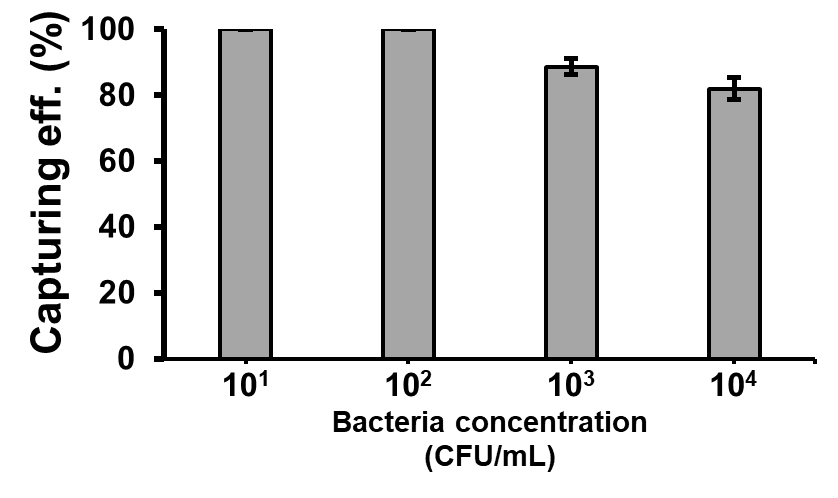


**Figure S2.** Efficiency of Van-PDA-MNPs in Capturing *Staphylococcus aureus* at Concentrations Ranging from 10^1^ to 10^1^ CFU/mL in Blood


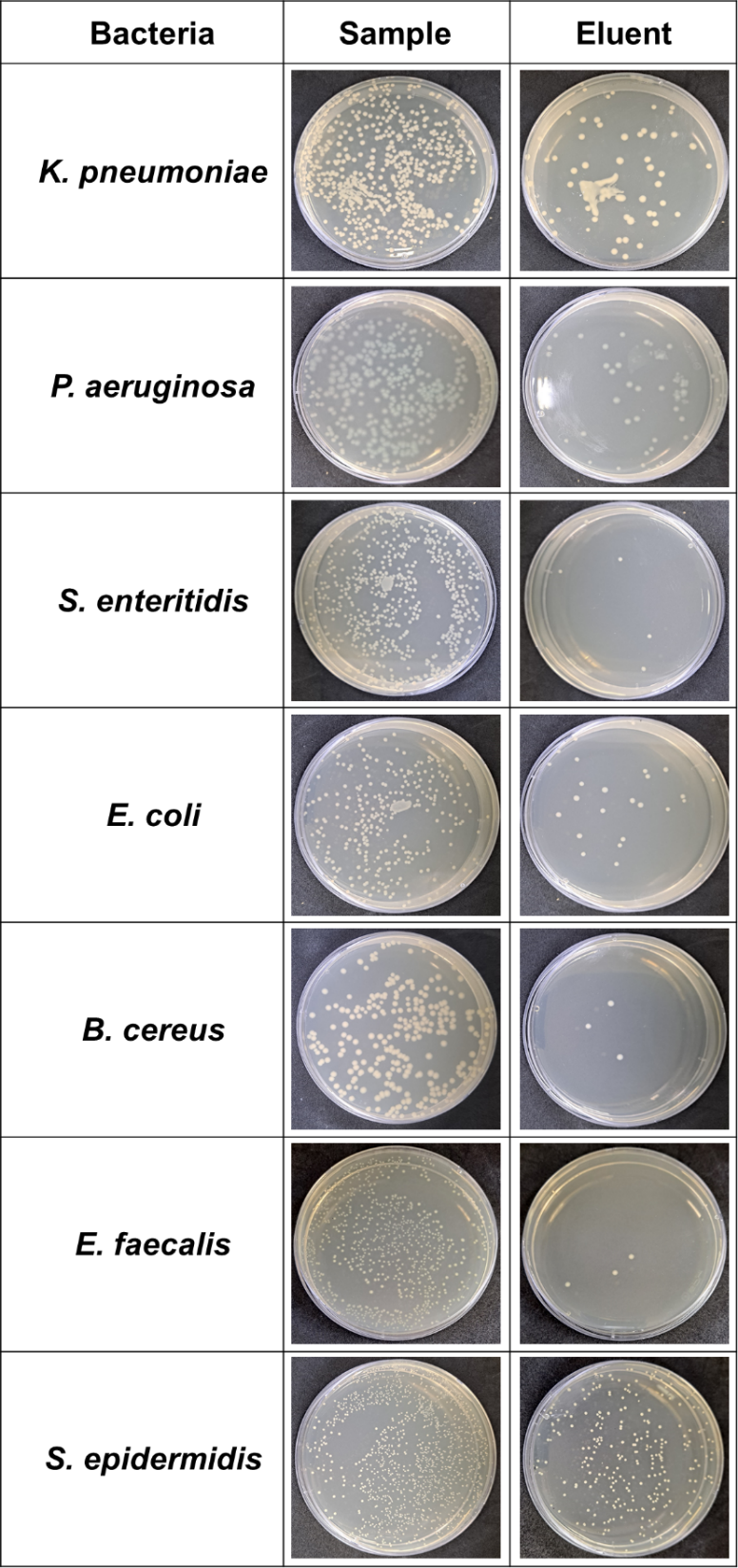


**Figure S3.** Representative colony counting images illustrating the efficacy of immunomagnetic separation with Van-PDA-MNPs and Al-PDA-MNPs within the automated system, providing visual evidence of successful pathogen isolation and capture.


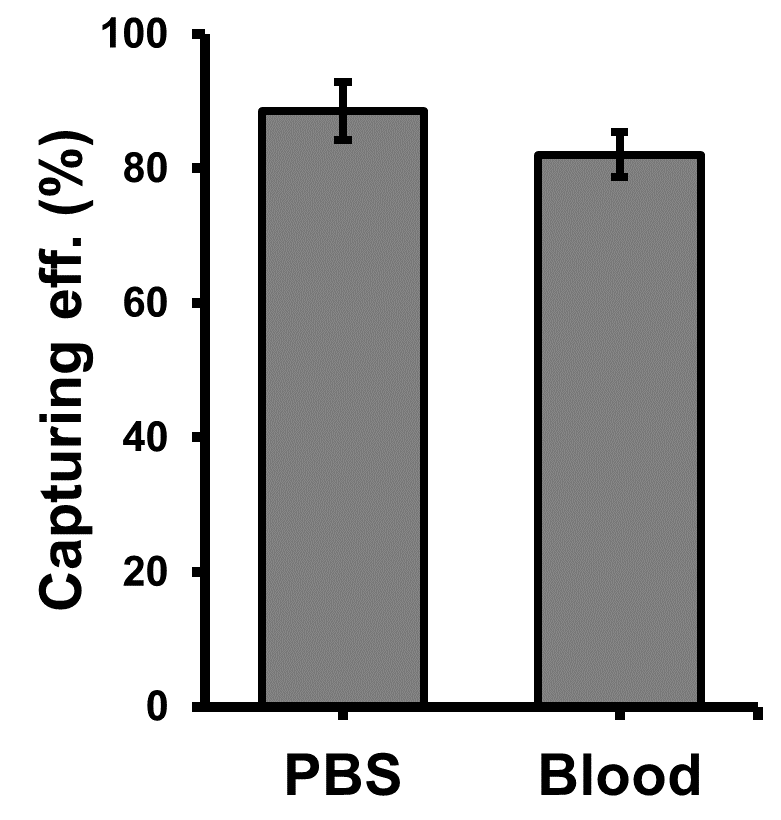


**Figure S4.** Capturing Efficiency of the AMSS with Van-PDA-MNPs for a Vancomycin-Resistant *Enterococcus* (VRE) strain (ATCC 52199) at 10^4^ CFU/mL.


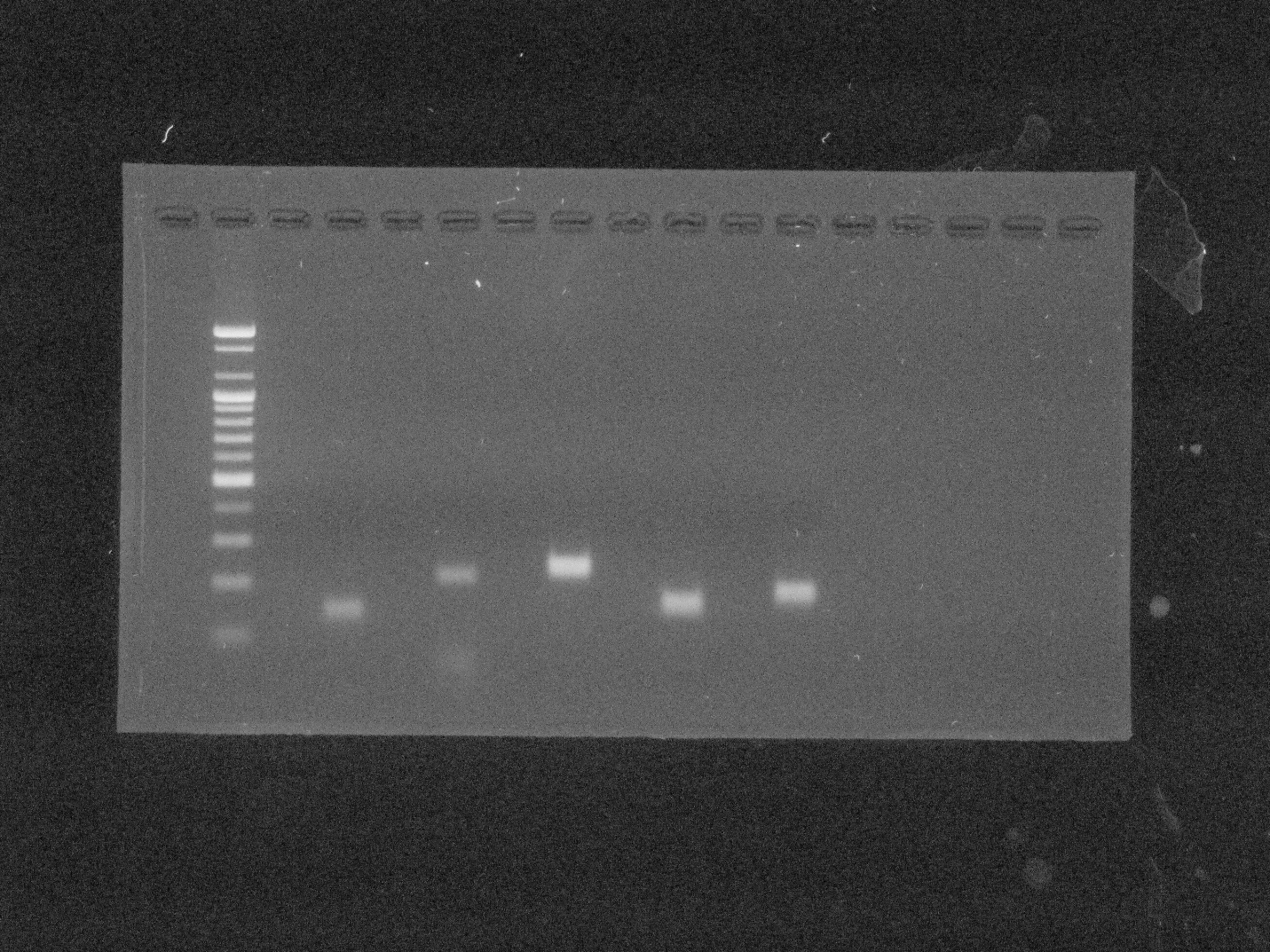


**Figure S5.** The gel electrophoresis image showcases DNA samples from Methicillin-resistant *Staphylococcus aureus*(MRSA), *Staphylococcus aureus*, *Bacillus cereus*, *Escherichia coli* O157:H7, and *Klebsiella pneumoniae*. Following PCR amplification, the products were resolved on a 2% TAE agarose gel at 100 V for a span of 30 minutes. For visualization, the UNOK-8000 gel documentation system by Korea Biotech (Daejeon, Korea) was used.


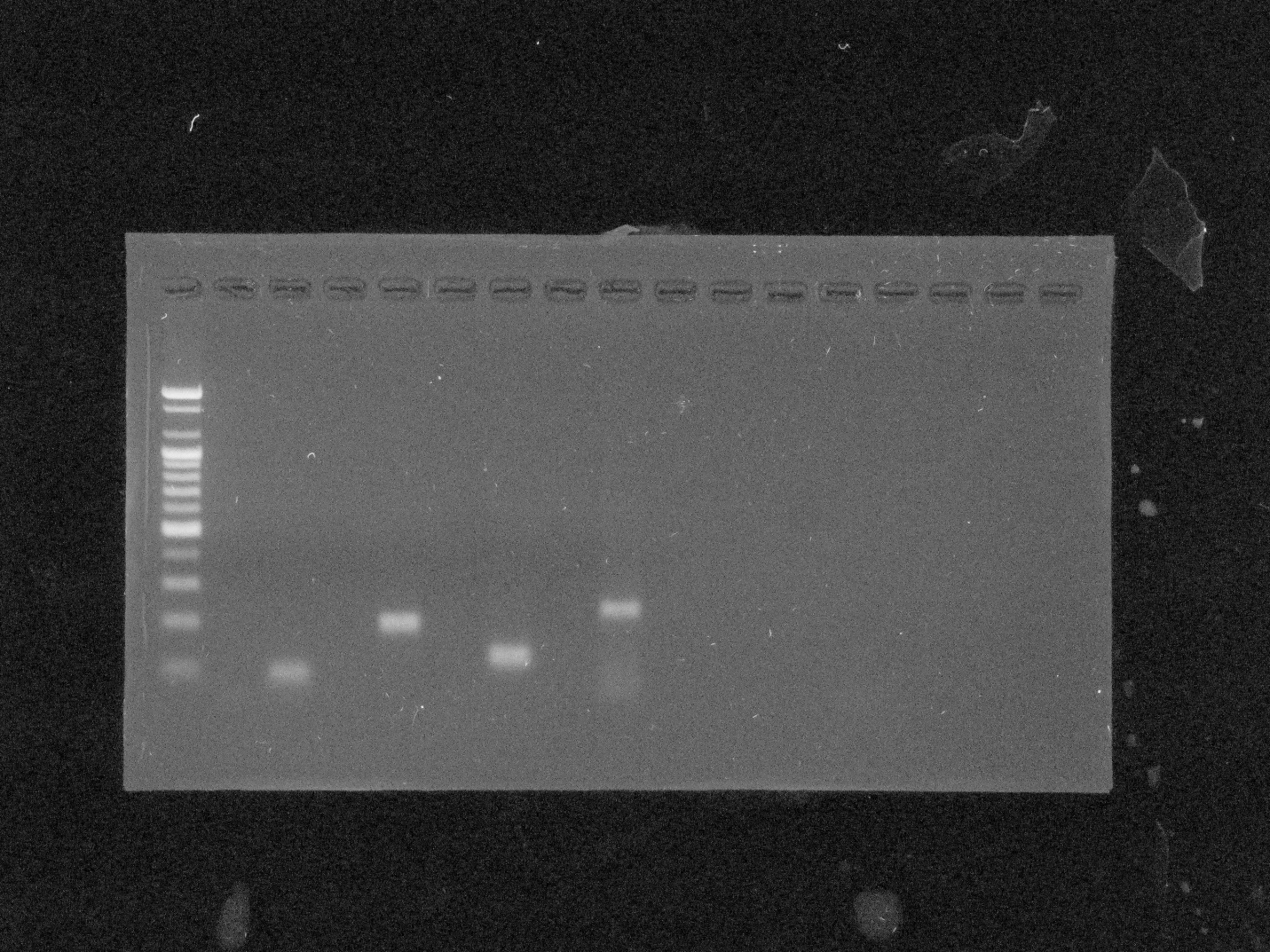


**Figure S6.** The gel electrophoresis image displays the DNA samples of *Enterococcus faecalis*, *Staphylococcus epidermis*, *Salmonella enteritidis*, and *Pseudomonas aeruginosa*. PCR products underwent electrophoresis in a 2% TAE agarose gel at 100 V for a duration of 30 minutes. The gel was visualized using the UNOK-8000 gel documentation system.
